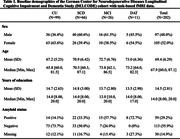# Longitudinal changes in brain activity during mnemonic discrimination over the course of Alzheimer’s disease

**DOI:** 10.1002/alz.095462

**Published:** 2025-01-09

**Authors:** Helena M. Gellersen, Hannah Baumeister, Frederic Brosseron, Anja Schneider, Klaus Fließbach, Annika Spottke, Stefan Teipel, Jens Wiltfang, Frank Jessen, Emrah Düzel, David Berron

**Affiliations:** ^1^ German Center for Neurodegenerative Diseases (DZNE), Magdeburg Germany; ^2^ German Center for Neurodegenerative Diseases (DZNE), Bonn Germany; ^3^ University Hospital Bonn, Bonn Germany; ^4^ University of Bonn Medical Center, Bonn Germany; ^5^ Department for Neurodegenerative Diseases and Geriatric Psychiatry, University Hospital Bonn, Bonn Germany; ^6^ Department of Psychosomatic Medicine, Rostock University Medical Center, Rostock Germany; ^7^ Department of Psychosomatic Medicine, University of Rostock, Rostock Germany; ^8^ German Center for Neurodegenerative Diseases (DZNE), Rostock Germany; ^9^ Rostock University Medical Centre, Rostock Germany; ^10^ Department of Psychiatry and Psychotherapy, University Medical Center, University of Goettingen, Goettingen Germany; ^11^ German Center for Neurodegenerative Diseases (DZNE), Goettingen Germany; ^12^ Neurosciences and Signaling Group, Institute of Biomedicine (iBiMED), Department of Medical Sciences, University of Aveiro, Aveiro Portugal; ^13^ Excellence Cluster on Cellular Stress Responses in Aging‐Associated Diseases (CECAD), Faculty of Medicine and University Hospital Cologne, Cologne Germany; ^14^ Department of Psychiatry, University of Cologne, Medical Faculty, Cologne Germany; ^15^ Institute of Cognitive Neurology and Dementia Research (IKND), Otto‐von‐Guericke University, Magdeburg Germany; ^16^ Department of Neurology, Otto‐von‐Guericke University, Magdeburg Germany; ^17^ Department of Psychiatry and Psychotherapy, Otto‐von‐Guericke University, Magdeburg Germany

## Abstract

**Background:**

Mnemonic discrimination tasks (MDTs) hold potential for early detection of memory changes in Alzheimer’s disease (AD). Object and scene processing tasks differently tap into memory networks vulnerable to early tau and amyloid pathology, respectively. We used an object and scene MDT to assess longitudinal effects of AD on distinct functional memory networks and investigate their potential as markers for different disease stages.

**Method:**

202 participants from the DELCODE study completed an object and scene MDT of highly similar objects and scenes during fMRI scanning each year from baseline to 36‐month follow‐up. Participants were classified as cognitively unimpaired (CU; Table 1) or having subjective cognitive decline (SCD), mild cognitive impairment (MCI), or dementia of the Alzheimer’s type (DAT).

**Result:**

The combined MCI/DAT group showed object and scene mnemonic discrimination impairment. SCDs only differed from CUs on scenes, but trended towards object discrimination decline over follow‐up. Cross‐sectionally, amygdala, BA35/36, anterior hippocampus, and parahippocampal cortex showed increased activity during successful discrimination of both scenes and objects. Entorhinal cortex, posterior hippocampus, and precuneus did so only for scenes, dovetailing with the framework of partially dissociable memory networks. Longitudinally, CUs showed increasing brain activity during object but not scene memory throughout the medial temporal lobe. SCDs showed a pattern of longitudinally increasing activity during scene memory coupled with decreasing activity during object memory in BA35, BA36, and hippocampus. Strikingly, the opposite was true for BA35 and entorhinal cortex in the MCI/DAT group.

**Conclusion:**

These results provide a first glimpse at longitudinal changes in brain activity during mnemonic discrimination throughout the AD continuum. Both neural and behavioural indices of the MDT may be sensitive to longitudinal disease effects. Future analyses will assess the complex relationship between disease effects on neurodegeneration, neural activity, and memory to better understand how early AD erodes memory and brain systems.